# The impact of routines on emotional and behavioural difficulties in children and on parental anxiety during COVID-19

**DOI:** 10.3389/frcha.2023.1114850

**Published:** 2023-12-13

**Authors:** Vera Lees, Rosie Hay, Helen Bould, Alex S. F. Kwong, Daniel Major-Smith, Daphne Kounali, Rebecca M. Pearson

**Affiliations:** ^1^ Child and Adolescent Mental Health Service, Gloucestershire Health and Care NHS Foundation Trust, Gloucestershire, United Kingdom; ^2^ Centre for Academic Mental Health, Population Health Sciences, Bristol Medical School, University of Bristol, Bristol, United Kingdom; ^3^ MRC Integrative Epidemiology Unit, Bristol Medical School, University of Bristol, Bristol, United Kingdom; ^4^ Population Health Sciences, Bristol Medical School, University of Bristol, Bristol, United Kingdom; ^5^ Centre for Academic Child Health, University of Bristol, Bristol, United Kingdom; ^6^ Faculty of Health, Psychology and Social Care, Manchester Metropolitan University, Manchester, United Kingdom

**Keywords:** routine, child behavioural difficulties, child emotional difficulties, parental anxiety, COVID-19

## Abstract

**Background:**

The Covid-19 pandemic and related public health measures, including lockdowns and school closures, have impacted on mental health of children.

**Aims and hypothesis:**

We hypothesised that there would be an association between maintaining a routine during lockdown and both lower emotional and behavioural difficulties in children and lower parental anxiety. Routine was taken as keeping to the same basic activities such as mealtimes and bedtimes. We also hypothesised that children of ‘keyworker’ parents would have fewer emotional and behavioural symptoms due to having maintained more normal routines. The key reason was that children of keyworkers still attended school or nursery and parents would have been getting up and coming home at the same times as pre-Covid. Keyworker status was defined as those whose work was essential to Covid-19 response, including work in health and social care and other key sectors.

**Methods:**

We used data from the Avon Longitudinal Study of Parents and Children (ALSPAC) to explore associations between maintaining a routine, and emotional and behavioural difficulties in children, using linear regression models. All eligible ALSPAC-G2 participants were sent the survey and the responders are representative of the eligible G2 population. We included measures of parental anxiety. We separately explored associations with having a keyworker parent. We used the Carey Infant Temperament Questionnaire and the Revised Rutter Parent Scale for Preschool Children to establish levels of emotional and behavioural difficulties. The measures were chosen to match previous waves in multi-generations in ALSPAC where they had been shown to be predictive of later mental health in children. The scales measure emotional and behavioural problems.

**Results:**

Two hundred eighty-nine parents completed questionnaires about their 411 children. Keeping a routine was associated with emotional and behavioural difficulty scores 5.0 points lower (95% CI −10.0 to −0.1), *p* = 0.045 than not keeping a routine. Parents who reported keeping a routine had anxiety scores 4.3 points lower (95% CI −7.5 to −1.1), *p* = 0.009 than those who did not. Children of keyworkers tended to have lower emotional and behavioural difficulty scores [−3.1 (95%CI −6.26 to 0.08), *p* = 0.056] than children of non-keyworkers. All models were adjusted for relevant potential confounders.

**Conclusion:**

Maintaining a routine may be beneficial for both child emotional wellbeing and parental anxiety, although it is also possible that lower parental anxiety levels made maintaining a routine easier. Being the child of a keyworker parent during lockdown may have been protective for child emotional wellbeing.

## Introduction

The Covid-19 pandemic affected populations across the globe, sparing no one (1). Families with small children had additional challenges not only to support their children with education, but also to explain, contain and navigate their children's emotions at times of global uncertainty and unpredictability. Many parents continued to work from home at the same time (2). Reactions to Covid-19 can present a once in a lifetime opportunity to find out about family life at times when families had to deal with health threats as well as the loss of social contacts (2). With such losses there are associated anxieties (3). Babore et al., observed that parents’ experiences during the lockdown, including prohibition of all relational contacts outside of the home and inability to access support, job insecurity and ill health of friends and loved ones, likely impacted parents’ distress levels, with a possible impact on children's well-being (4).

Parenting stress has been shown to impact on the emotional wellbeing of their children, with association between maternal anxiety and psychological difficulties in their children (4–6). Parents with higher perceived parenting stress were less sensitive to their children's needs and showed more dysfunctional interactions with their children (4). Similarly, Morelli et al. found that parental distress impacted on the emotional regulation of their children and that this was magnified in children with a chronic health condition and their parents (7, 8). Masi et al. examined the impact of the pandemic on children with neurodevelopmental disabilities, and found that these children were exercising less, had increased television and digital media use, had a poorer diet and poorer sleep quality during the Covid-19 pandemic (9). We can infer that routine had changed for these families during the pandemic (9). From this previous research, it is understandable that pressures from a pandemic may result in increased parental stress within a family system and subsequently impact on the emotional wellbeing of children and young people during this time.

However, Co-Space Study, set up to increase understanding of how families coped throughout the Covid-19 pandemic, in one of their earlier reports surveying parents in August 2020 found that lockdown was beneficial to young children despite childcare challenges. According to parents’ reports children's emotional problems did not increase or decrease during the month of the survey. Additionally, employed parents/carers, but not those who were unemployed, reported a reduction in their child's behavioural and restless/attentional difficulties over the one-month period, however the changes were subtle (10).

Overall accumulating global evidence suggests that the Covid-19 pandemic and associated public health measures including self-isolation, lockdowns and school closures have had a significant negative impact on the mental health of children and young people (11–13). There have been increases in mental distress, eating disorders and self-harm amongst children and young people (13). Data on younger children is lacking, but our own previous work indicates a rise in emotional and behavioural symptoms in primary school aged and younger children (14). It is important to understand factors implicated in such rises, particularly those which could be protective for children and young people. A better understanding of protective factors gives potential to target appropriate intervention to support the mental health of younger children in the event of future pandemics or Covid-19 suppression measures.

One possible protective candidate, which evidence suggests is helpful in children’s daily life, is maintaining routine (17). Routine helps us plan, deliver, predict what to expect, and may protect us from anxiety during stressful times (16, 17). Structure and consistency are important for children; for example, keeping regular mealtimes and bedtimes can help children feel safe and secure (17). Predictability and familiarity are especially important for children who are still learning and growing, and has also been shown to be important in the context of adolescent mental health (18).

Routines have also been suggested as an area of focus for intervention among families living in chaotic households with young children who present with behavioural problems and bedtime resistant behaviour (19). The Covid-19 pandemic and responses to mitigate its effect significantly disrupted routine for both children and their parents. Family routines were upset by the disruption of engagement with most activities, educational, work, social and even basic established routines such as shopping (1, 2). Babore also highlights that the social distancing measures associated with the pandemic and related changes to childcare routines meant many mothers developed new routines and set new limitations with their children (4).

The UK entered its first national lockdown on 23rd March 2020. Lockdown meant that schools were closed to children, other than children of keyworkers and those considered vulnerable. The British government specified categories of these keyworkers whose work was essential to Covid-19 response, including work in health and social care and other key sectors (20). Children defined as vulnerable included those on child protection plans with local authorities and those who struggled to engage with education remotely (18). For many children during lockdown, their normal routine completely disappeared: their schooling changed, their social contacts, hobbies and childcare changed; and many were no longer able to be in contact with grandparents and extended family. Parents’ routines also changed, with some multitasking home-working with home-schooling or childcare, and others being furloughed from employment. Restrictions were partially lifted with children in nursery, Reception, Year 1 and Year 6 able to return from 1st June 2020. From 15th June 2020, Year 10 and Year 12 were permitted limited contact to help prepare for exams.

One study has found that the pandemic has affected behaviours of children and adolescents, in that it has led to decreased physical activity and disrupted sleep patterns (21). The NHS Digital Survey of Mental Health of Children and Young People also demonstrated significant changes for children and young people in terms of their education and usual activities due to the Covid-19 pandemic (22).

In addition to routines supporting children's mental health, childcare availability and the associated routines may also impact parental mental health, including anxiety (17). Research shows that parents’ individual distress has been associated with poor mental health in children. Specifically, depression and anxiety in mothers were shown as risk factors for depressive and anxiety symptoms in children (4). Previous research into the consequences of being quarantined during the H1N1 influenza outbreak, found that stressful isolation resulted in increased psychological distress in parents which subsequently impacted on the wellbeing of their children (23).

As the previous research suggests that routine has a protective role for children's mental health, we hypothesised that keeping to routine would be associated with fewer emotional and behavioural difficulties in children during unprecedented public health crisis and we examined the associations between routine and emotional and behavioural difficulties. As evidence suggests routine can impact and reduce anxiety in parents, we hypothesised an association with routine and lower parental anxiety. We explored the impact of Covid-19 on children of keyworkers. Keyworker parents were more likely to have maintained their usual routine, therefore we hypothesised that being a keyworker parent would be associated with fewer emotional and behavioural problems in children. Their children were able to continue attend educational setting during lockdown, therefore they may have been less affected by routine disruption. However, children may have been more worried both about themselves catching the virus and their parents being at risk of Covid as frontline workers. To demonstrate an association between routine and a child and parental stress this study will highlight that routine was associated with reduced stress in both a child and a parent and thus may be a mutual protective factor.

## Method

The Avon Longitudinal Study of Parents and Children (ALSPAC) is an ongoing population-based study that recruited pregnant women residing in Avon in the south west of England with expected delivery dates between 1st April 1991 and 31st December 1992 (24–26). The cohort consists of mothers and their partners (G0) associated with 15,454 pregnancies, resulting in 15,589 foetuses (G1) of which 14,901 were alive at 1 year of age. In 2012, ALSPAC began recruiting and collecting data on the next generation, G2, the children of the G1 participants and grandchildren of the originally recruited G0 women (also known as the ‘children of the children of the 90s’). G2 participants can join the study at any time (from early pregnancy onwards), through an open cohort (27). As with the original study, data are being collected from both parents (at least one of whom is a G1 participant) and their children. The study website contains details of all data available through a fully searchable data dictionary (http://www.bristol.ac.uk/alspac/researchers/our-data/).

In June 2020, G1 parents (mean age ∼28 years) completed a questionnaire about each of their G2 children (27), and a questionnaire about themselves (28). These questionnaires were completed early in the Covid-19 pandemic (between 26th May and 5th July 2020), as part of ALSPAC's Covid-19 data collection strategy (29). Study data were collected and managed using REDCap electronic data capture tools hosted at the University of Bristol (30). REDCap is a secure web application for building and managing online surveys and databases.

### Measures of routine

Participants were asked whether they kept a similar routine (e.g., bedtime, mealtimes) to how things were before the official lockdown was announced on 23rd March 2020, with response options “no, not at all”, “yes, a bit”, “yes, a lot” and, “yes, completely”. The latter two categories were combined into one due to low frequency of participants endorsing the original separate categories, resulting in three categories in total.

### Keyworker status

G1 parents were asked whether they were a keyworker, or whether their work had been classified as critical to the Covid-19 response, with options “yes”, “no”, or “don’t know”.

### Measures of child emotional and behavioural difficulties

Parents who participated in the Covid-19 survey completed one of two assessments regarding their child's feelings and behaviour since the lockdown. The assessment version depended on the child's age. Parents of children less than 36 months old (henceforth referred to as younger children) completed the mood and distractibility subscales of the Carey Infant Temperament Questionnaire (ITQ) (31). Parents of older children (ages 36 months and older) completed the Revised Rutter Parent Scale for Preschool Children (32). Measures which were the same as, or as close as possible to the two used during the pandemic were selected from assessments completed by G1 parents before the pandemic. The same standardised scores were used (32).

### Measures of parental anxiety

Parental anxiety was measured using the GAD-7, a 7-item questionnaire with scores ranging between 0 and 21, with scores >10 indicating probable generalised anxiety disorder (33).

### Covariates

The following covariates were included: maternal age in years (continuous variable); parity (continuous variable); maternal education (binary variable: GCSE equivalent or below, or A level or above); child’s age in years (continuous) and child’s gender (binary).

### Statistical analysis

Linear regression models were employed, with standardised emotional behavioural problems as the outcome, and routine and keyworker status as separate categorical exposure variables. Analyses were adjusted for relevant confounding variables for the specific association under investigation considering the relationship with the exposures and outcomes. Relevant variables included child age, gender and maternal education, maternal age, parental anxiety and parity. Amongst the ALSPAC-G2 children, there were 122 sib-sets, and therefore we accounted for clustering using robust standard errors, clustered on parent ID (34–36). We also ran linear regression models of the association between maternal anxiety and routine. The relationship between child emotional difficulties and parent anxiety is likely to be complex and bi-directional therefore we did not include the two variables in the same model and rather in this work present the associations with routine. All analyses were adjusted for child age, gender and maternal education, maternal age and parity and were conducted in Stata v.15/MP. Linear regression was chosen due to the simple nature of the analysis question and interpretability of the coefficients. The coefficients are the estimated effects.

Mixed effects linear regression would have been an alternative way of modelling this data and would lead to more efficient estimates albeit with slightly different interpretation. The implemented approach leads to the most conservative estimates for standard errors (34–36).

The design in relation to the analysis is cross sectional, the exposure and outcome used in the paper are measured at the same time.

## Results

In total, 411 G2 children had questionnaire data, completed by their 289 parents, 234 parents had complete data. This resulted to 328 out of 411 children having complete data. Because there are multiple different questionnaires with different information 83 were missing at least one answer on one of the surveys. Of these 411 G2 children, 214 (52%) were male. The median age for G2 children was 34 months (IQR 16–71 months) (28). Of the 411 children, 404 had complete data on keeping a routine. Of those with data, 41 (10%) did not keep the same routine; 182 (44%) kept the same routine “a bit”, 181 (44%) kept the same routine either “a lot” or **“**completely”.

Of the 411 children, 328 had complete data including for all covariates. The analyses reported here concern the children with complete data. The reason for incomplete parental data is that at the time when the first Covid-19 survey was rolled out, the participating parents’ children were not old enough to have participated in the scheduled ALSPAC clinics where detailed assessments and demographic data are collected (11). There were no differences in the difficulty scores between the children excluded because there was no parental data and those included in the adjusted analyses. This reduced statistical power but there was no evidence that those with missing data were different in key variables (for example they were not higher on anxiety scores), therefore there is unlikely to be any systematic biases.

### Routine and emotional and behavioural difficulties in children

**Figure 1:**
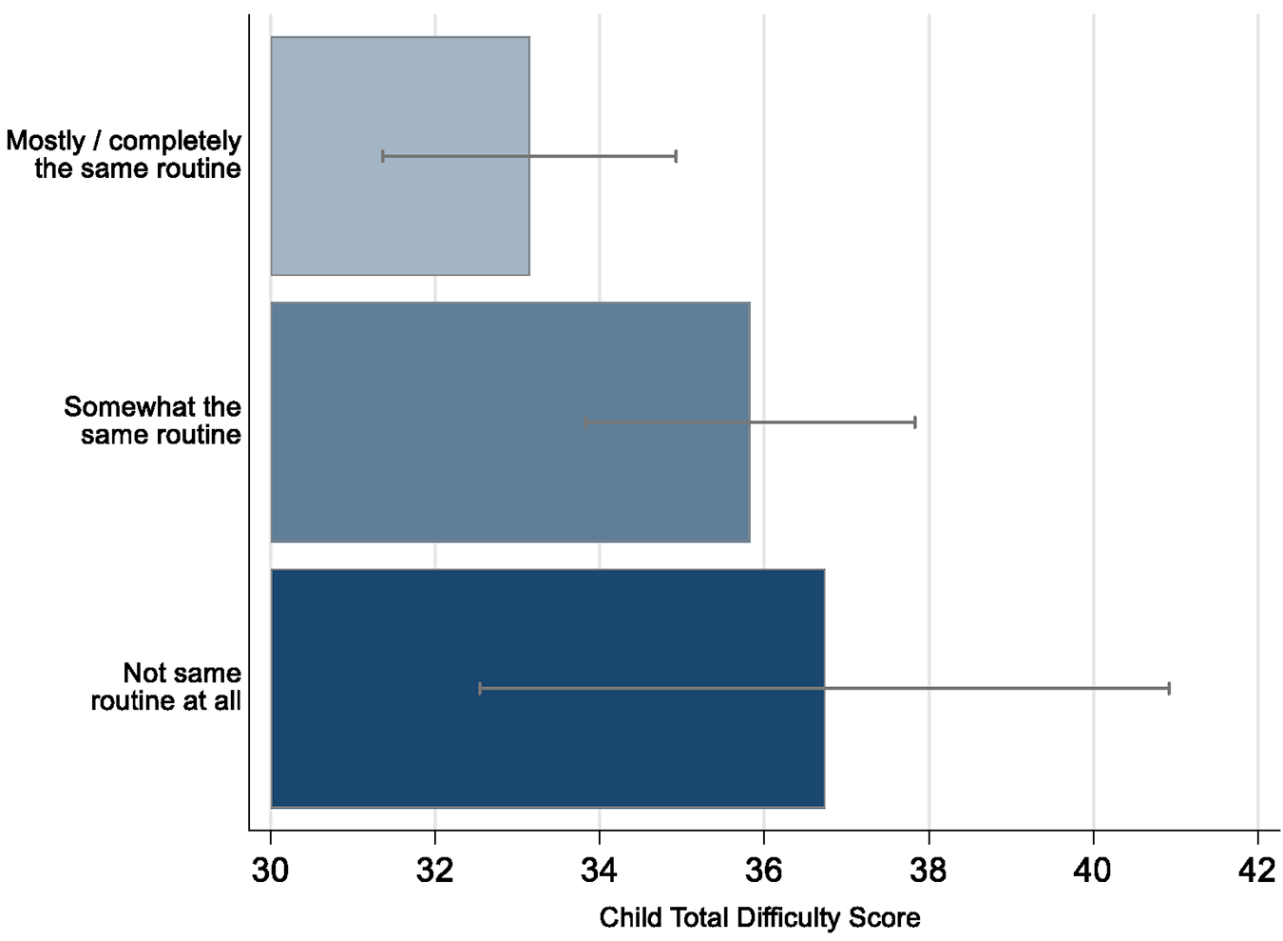
COVID-19 pandemic related total difficulty scores in children by keeping routine in ALSPAC. The error bars represent 95% CI.

We found a linear pattern between the extent of keeping routine and lower emotional and behavioural difficulties in the children (Figure 1). This association was not altered by clustering by maternal ID, or by adjusting for child gender, maternal parity, maternal and child age, maternal educational background (Table 1; higher difficulties scores indicate more emotional and behavioural difficulties). Compared to families where no routine was kept, children of those parents reporting keeping “a lot” or “completely” the same routine to before lockdown had emotional and behavioural difficulty scores 5 points lower (95% CI −10.02 to −0.12; *p* = 0.045). There was less evidence of a difference in emotional and behavioural difficulties between those reporting keeping the same routine “a bit” in comparison to those not keeping the same routine at all.

**Table 1:** Linear regression comparing the association between the extent of routine and child difficulties score.

Routine	Linear regression coefficient for emotional difficulties score (unadjusted)	Linear regression coefficient for emotional difficulties score (adjusted)^a^
Not at all	REF	REF
A bit the same	−2.13 (95% CI −6.77 to 2.51) *p* = 0.368	−1.59 (95% CI −6.31 to 3.13) *p* = 0.509
A lot/completely the same	−5.62 (95% CI −10.39 to −0.86) *p* = 0.021	−5.01 (95% CI −10.02 to −0.12) *p* = 0.045

^a^ accounting for clustering according to mother id and adjusted for child gender, number of children, maternal and child age, educational background.

### Routine and parental anxiety

**Figure 2:**
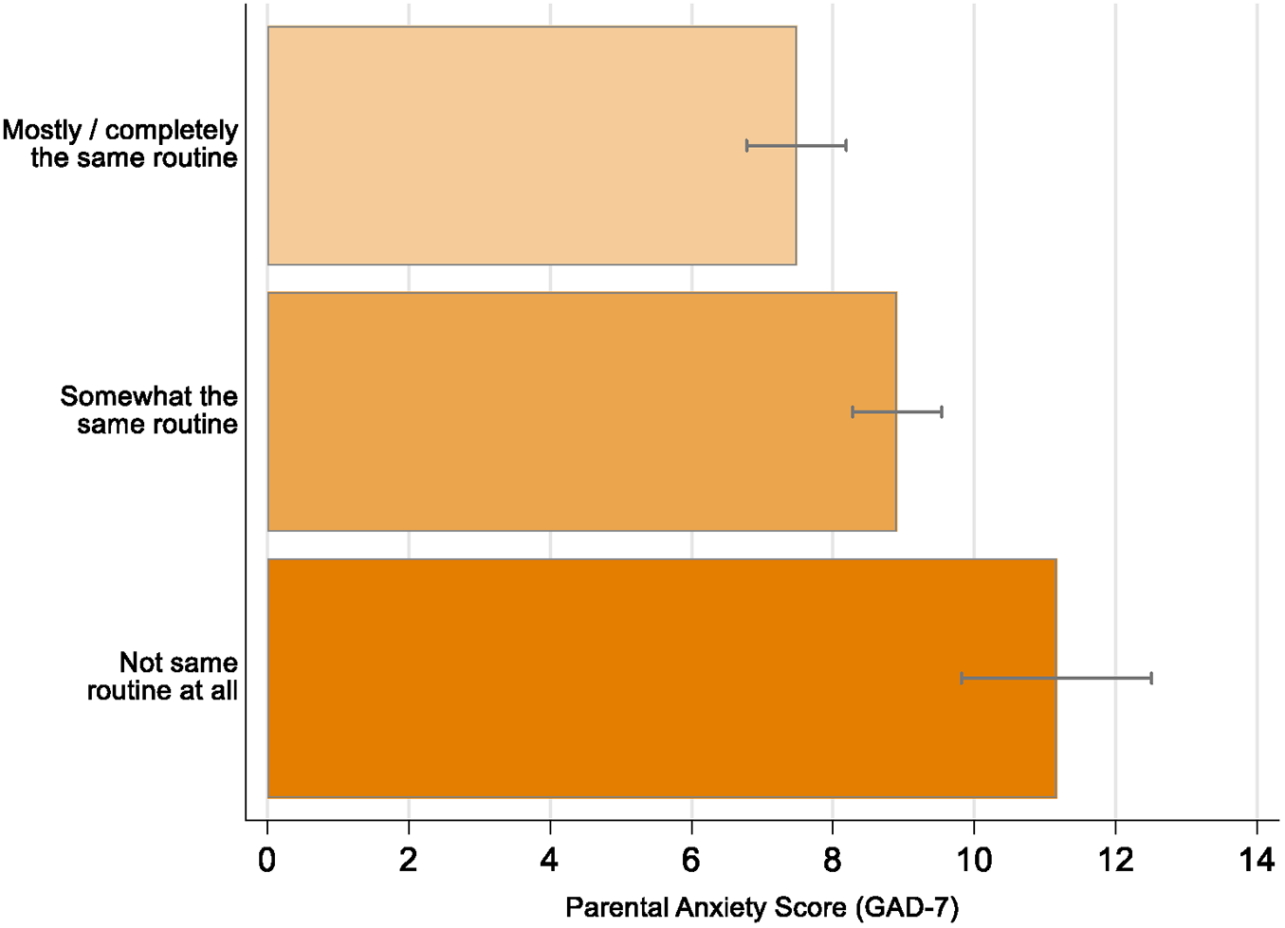
COVID-19 pandemic related parental anxiety score by keeping routine in ALSPAC. Error bars represent 95% CIs.

Parents of 314 G2 children had data on anxiety measured in the second Covid-19 questionnaire and on childcare routines. Parents who kept to a routine were less anxious than those who did not, again finding a linear pattern (Figure 2). A mean GAD score of 11.2 (95% CI 9.82–12.51) (indicating probable generalised anxiety disorder) was found in the group that did not follow a routine at all. Parents who kept to the same routine “a bit” had a mean GAD score of 8.9 (95% CI 8.28–9.54). Those parents who followed “a lot/completely the same” routine had mean GAD score of 7.5 (95% CI 6.78–8.20). Table 2 shows the results of the linear regression analyses describing the association between routine and parental anxiety. The magnitude of change in anxiety scores is considered clinically meaningful, with such changes in symptoms of similar magnitude being associated with improvement following clinical interventions like Cognitive Behavioural Therapy (CBT) and perceived feelings of feeling better (37).

**Table 2:** Linear regression comparing the association between the extent of routine and maternal anxiety scores.

Routine	Linear regression coefficient for maternal anxiety score (unadjusted)	Linear regression coefficient for maternal anxiety score (adjusted)^a^
Not at all	REF	REF
A bit the same	−3.46 (95% CI −6.02 to −0.91) *p* = 0.008	−4.43 (95% CI −7.60 to −1.30) *p* = 0.005
A lot/completely the same	−4.30 (95% CI −6.86 to −1.75) *p* = 0.001	−4.30 (95% CI −7.5 to −1.10) *p* = 0.009

^a^ accounting for clustering according to mother id and adjusted for child gender, number of children, maternal and child age, educational background.

In the model taking into account maternal and child age, gender, maternal education and parity there was evidence that, compared to families where no routine was kept at all, parents who kept “a lot/completely the same routine” to before lockdown, had anxiety scores on average 4.3 points lower (95% CI −7.5 to −1.1; *p* = 0.009). Those who kept the same routine “a bit” had parental anxiety scores on average 4.4 points lower (95% CI −7.60 to −1.30; *p* = 0.005) than those who did not keep the same routine at all (Table 2).

### Keyworkers

147 children had one or both parents who were keyworkers; and 160 had one or both parents who were not keyworkers. Parents were counted more than once if they had more than one child.

**Figure 3:**
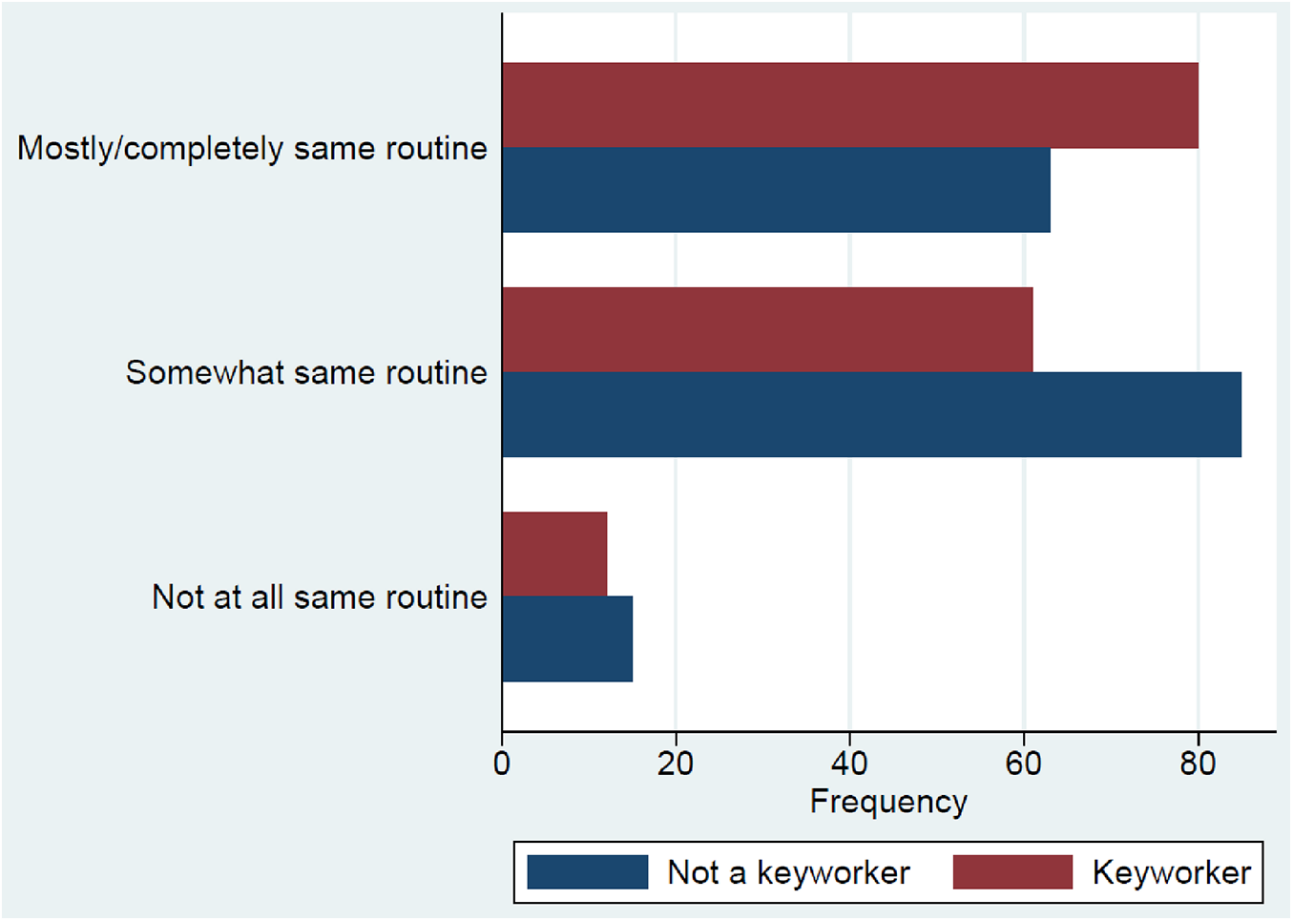
Routine kept by keyworker and non-keyworker parents.

Children with at least one keyworker parent were more likely to have kept similar routine to before lockdown than children with no keyworker parent {Figure 3, [Chi^2^ (2) 22.13, *p* < 0.001]}, whilst they were less likely to report they kept to ’somewhat same routine’ and no differences were apparent for ‘not at all same routine’.

**Figure 4:**
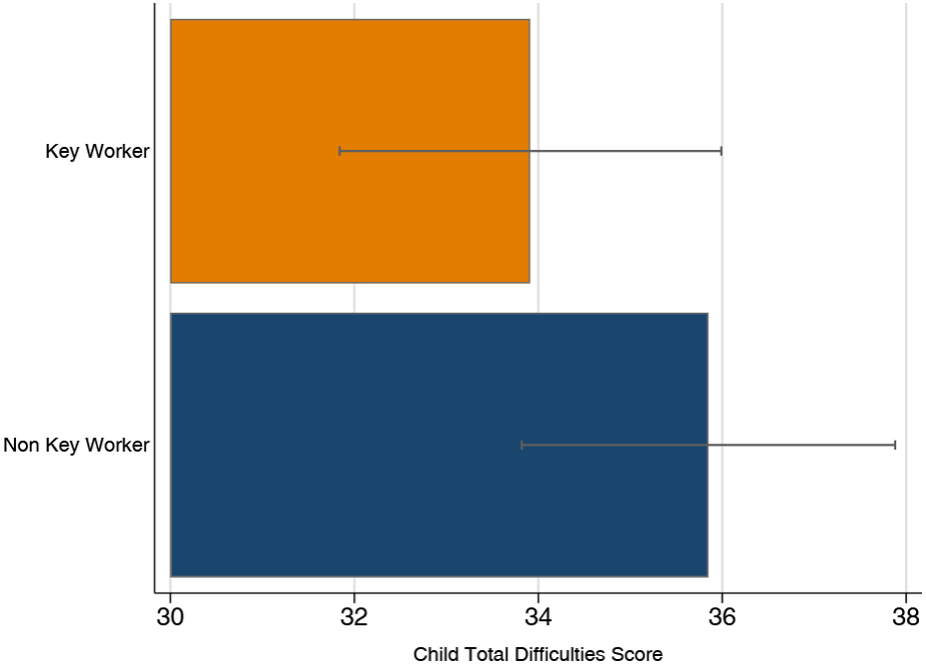
Difficulty scores for children of keyworkers and non-keyworkers. Error bars represent 95% CIs.

Children of keyworkers were also reported to have fewer emotional and behavioural difficulties during lockdown than children of non-keyworkers (Figure 4). After adjusting for maternal and child age, maternal education, gender, and parental anxiety, keyworker children scored on average 3 points lower (95% CI −6.26–0.08; *p* = 0.056) than children of non-keyworkers. However, the evidence against an effect of this magnitude occurring under the null hypothesis is weaker, relative to some of our other results (Table 3).

**Table 3:** Linear regression comparing the association between parental keyworker status and child difficulties scores.

	Linear regression coefficient for emotional difficulties score (unadjusted)	Linear regression coefficient for emotional difficulties score (adjusted)^a^
Not keyworker	REF	REF
Keyworker	−2.67 (95% CI −5.90–0.539) *p* = 0.102	−3.09 (95% CI −6.26–0.08) *p* = 0.056

^a^ accounting for clustering according to mother id and adjusted for, number of children, maternal and child age, educational background and anxiety.

## Discussion

### Strengths and limitations

We report evidence of an observed linear pattern between the extent to which parents perceived that routine stayed the same and lower emotional and behavioural difficulties scores in children, as well as lower anxiety in parents. Our results are consistent with previous studies that link parental mental health to the emotional wellbeing of children during Covid-19 (4, 22). It is also consistent with research reporting protective effects of routine on children's wellbeing outside of the pandemic (15, 38).

The mechanisms to explain the association remain unclear from this research alone. However, some speculation is possible. The onset of the Covid-19 pandemic and public health responses to it brought about significant changes for society, not least for the family unit and routines for families. Firstly, as descried by Lebow, changes in family dynamics as a consequence of the pandemic are likely to present multiple challenges due to suddenly unpredictable daily activities (3). Practical changes in times that would be normally structured around work and school were suddenly changed. In this explanation we argue that the change in routine impacted both parent and child independently to each other.

Secondly, the routine and structure may have been a consequence of less anxiety in the parent, their personality traits or indeed their own coping strategies to manage the pandemic. It is well known, Babore showed, that the effect of mothers’ individual distress on children's depression was mediated by mothers’ parenting stress (4). Given that maternal anxiety, routine and child emotional symptoms were all measured around the same time and it was not possible to track the precise time course of the impact on family dynamics both explanations remain possible. Either way our findings highlight that keeping a routine may be linked to positive outcomes, whether or not that is directly felt by the child or via improving mothers’ mood. Indeed, it is increasingly understood that emotional symptoms in families are transactional, bi-directional and in reality, hard to disentangle. Thus, although the mediating role of parenting stress on child mental health can be examined in longitudinal stuides (4), adjusting for maternal anxiety here would lead to uninterpretable results.

It is also not possible to draw firm conclusions on what specific aspect of routine may be significant in terms of being protective for children from this research. However, in this paper, routine refers to bedtimes and mealtimes, not wider routines like childcare settings, schooling, exercise/physical activity, and socialising and therefore it may be hypothesised that even simple and predictable habits such as times of eating and sleeping are linked to positive outcomes.

There is little previous research that has considered how being a ‘keyworker’ parent may affect children's emotional wellbeing. This is likely, in part, due to the fact that the term keyworker appears to be unique to the United Kingdom and became a specific concept during Covid-19 to consider ‘essential’ workforce in the short term. However, our findings that keyworker parents were more likely to keep routines, and a suggestion that emotional and behavioural difficulties scores were lower in children of keyworkers than those of non-keyworkers was in line with our hypothesis that parents’ work schedule continued without change. In addition, in the UK keyworker children were able to attend their education provision, albeit with an amended education programme, they also maintained more friendships and social routines. There was also a greater sense of being valued and purpose of those families who were keyworkers and this could provide a further explanation given that sense of purpose is associated with lower stress levels (39).

The data was from self-report which may not have reflected reality, for example, a parent may have perceived their routine was similar but actually objective data may have shown they were not. For example, getting up later or perhaps eating at different times. Passive data collection in phones could be a more objective way to study routine in the future, although this was not feasible in this study.

Whilst we have described evidence from other research that the lockdown has had a negative impact on the emotional wellbeing, there have also been some studies that found that the mental health and wellbeing of children and young people actually improved during the lockdown period. This was particularly true for young people who had poor mental health pre-pandemic and those who had difficulties with school and peer relationships at school (40, 41). A survey carried out by Pavlopoulou in 2020, whilst only a small cohort, demonstrated that while lockdown may have increased pressures for some autistic young people, others found that having less social pressure, lesser sensory challenges, and having more available time to spend on their own interests, was actually beneficial (42). However, they did identify that less support and lack of adapted work online, added to challenges for the families of autistic young people. This indicates that an individualised approach to stressful life events would be advisable.

This is a novel study looking at the effect of Covid-19 pandemic on younger children, using longitudinal pre and during pandemic data from the ALSPAC cohort.

The median age of the children in our study was 34 months. It is possible that the results may have been different if our cohort had included older children, as their increased awareness and understanding of Covid-19 and their keyworker parents being exposed to the virus could mean that their anxiety would be higher, even if their routines were maintained. Including adolescents in this study was not possible due to the demographic of the G2 cohort. In addition, it is possible that the relatively young age of the parents means that they have less stability in jobs and therefore this may contribute to how well routine could be maintained.

### Generalisability and attrition

The population in the sample is limited in ethnic diversity and geographical spread, which may limit the generalisability of our results to other populations. It was not possible to mitigate this, as the cohort have self-selected into the ALSPAC study. We acknowledge that there may be differences in the results if the demographic of the participants was more diverse. In addition, ALSPAC has incomplete recruitment and loss to follow-up; the current recruitment at a relatively young age and the focus of analyses in cross-generational effects are more specific to ALSPAG-G2 (25). Furthermore, whilst we have attempted to adjust for factors that may confound the relationship between routine and emotional and behavioural difficulties, it is possible that residual confounding from unmeasured variables may affect this apparent relationship.

### Reverse causality

The cross-sectional nature of this work means that, although we have described an association between keeping routine and fewer behavioural and emotional difficulties in children, we are unable to determine the direction of causality, i.e., we cannot conclude that keeping to routine leads to lower emotional and behavioural difficulties, since it is also possible that children having fewer emotional and behavioural difficulties makes it easier to follow a routine. Furthermore, the phrasing of the questions on “routines” compares current routine to pre-lockdown routine. If there was little routine pre-lockdown, then answering “yes, completely” to maintaining routine during lockdown could still indicate lack of routine.

### Measurement

Importantly, child emotional and behavioural difficulties are reported by the parent. This is potentially subject to reporter bias. For example, where a parent is struggling with their own mental health, a child's usual behaviour may be perceived as more challenging because the parent is finding their child's behaviour more difficult to manage rather than because the child's behaviour has changed significantly (46). There is conflicting empirical evidence on the existence of systematic bias in the rating of child's behavioural and emotional problems and this is affected by the parent's mental health characteristics. However, previous work and sensitivity analyses using the same sample, did not find any evidence to support this (44). Our previous work demonstrated that while there are differences between parent and teacher or parent and child report of emotional symptoms, these differences are not associated with maternal depression or anxiety and therefore it is unlikely to be systematically biased by maternal depression or anxiety (45).

This is further supported by other studies suggesting that biases are negligible and that any differences in behaviours linked to reporters maybe the result of cross-situation differences in child behaviour, differential abilities of parents in recognising psychological problems and/or direct and indirect genetic confounding (46).

Whilst we have acknowledged the potential bias, though negligible, parent report measure was still felt to be the most appropriate way to gather a large amount of information for this research as there would not be a reliable way to gather information from the children themselves, due to their young age. Parents are the most appropriate people to report on observed differences in their children pre pandemic and during the pandemic in this cohort.

### Conclusions, implications and future directions

As Ofsted report emphasizes, school closures have resulted in children regressing in their learning as well as increasing mental health distress, eating disorders and self-harm (13, 46). Our research suggests an increase in emotional and behavioural difficulties, associated with difficulties maintaining childcare routines during the same period. We demonstrate that routines are associated with good mental health, both in children and in parents. Whilst we cannot confirm the direction of causality from these data alone, if causal, this would suggest that interventions to facilitate routines could help prevent difficulties. Most parenting advice regarding lockdown mentions keeping routine and this research supports that advice. Since it is possible that parents with higher levels of anxiety are less likely to have kept to the same routine, it may be that families need different levels of support to achieve this aim. Furthermore, it is possible that parents who are able to maintain a routine are more likely to be of the temperament to complete questionnaires which may introduce a reporting bias.

Our results also suggest that it is important to support parents’ mental health. Keeping childcare provision open may also mean that routines can be more consistent and may reduce anxiety for parents who are juggling work and childcare responsibilities. During future pandemics, public health services should support parents, and particularly mothers, in reducing individual distress and parenting stress, as these are also associated with children's depression (4).

Due to the breadth of data collection capturing multiple exposures, we were not able to identify specific roles of keyworker parents, or whether they started a new role as a keyworker during the pandemic. This would be an interesting area to consider in future research. This is a large cohort survey, which has highlighted the potential role of routine on emotional wellbeing. It has generated direction for more in depth research into specifics of routine, which may be captured by future studies using validated questionnaires to look at bedtime routine, for example.

In the event of a pandemic in the future, parents might benefit from being given information that would support them in maintaining a routine. For example, an online advice and guidance page with suggestions on how to achieve this. Similarly, information that is tailored for families of children with specific needs, such as neurodiversity or learning disability, would be beneficial for parents in these difficult circumstances. Now that online platforms are readily available and familiar to many, support programmes could be developed to ensure parents feel supported with helping their family keep to a routine.

We conclude that maintaining routine may be beneficial for children's emotional wellbeing as well as parental anxiety. Thus, maintaining routine may be a mutually protective factor. Further research exploring the direction of causation may provide us with greater understanding. Being the child of a keyworker parent during lockdown may have been protective for child emotional wellbeing. This may be reassuring to keyworkers, however further work to replicate this finding is needed. Whilst this study focused on the importance of maintaining routine during a pandemic, it is reasonable to surmise that during any time of stress, such as moving a house, an illness in a family or other significant change in life, maintain usual routine will be beneficial to emotional wellbeing for both children and parents.

## Data Availability

The original contributions presented in the study are included in the article/Supplementary Material, further inquiries can be directed to the corresponding author.
